# Investigation of the idiosyncratic hepatotoxicity of *Polygonum multiflorum* Thunb. through metabolomics using GC-MS

**DOI:** 10.1186/s12906-021-03276-4

**Published:** 2021-04-12

**Authors:** Yan Lin, Rong Xiao, Bo-hou Xia, Zhi-min Zhang, Chun Li, Ping Wu, Duan-fang Liao, Li-mei Lin

**Affiliations:** 1 College of Pharmacy, Hunan University of Chinese Medicine, Changsha, 410208 China; 2 China Institute of Chinese Materia Medica, China Academy of Chinese Medical Sciences, Beijing, 100700 China

**Keywords:** *Polygonum multiflorum* Thunb., Idiosyncratically hepatotoxic components, Tetrahydroxystilbene glucoside, Mechanism of idiosyncratic hepatotoxicity, Differential biomarkers

## Abstract

**Background:**

The idiosyncratic hepatotoxicity of *Polygonum multiflorum* (PM) has attracted considerable interest, but the idiosyncratically hepatotoxic components and endogenous metabolite changes resulting from idiosyncratic hepatotoxicity of PM are not well understood. The aim of this study was to identify the idiosyncratically hepatotoxic components and potential endogenous metabolic biomarkers for PM-induced liver injury.

**Methods:**

Serum biochemical indicators and hematoxylin and eosin (H&E) staining were evaluated to identify pathological changes. Gas chromatography/mass spectrometry (GC-MS) was performed to identify changes in metabolic biomarkers. Orthogonal projection to latent structures discriminant analysis (OPLS-DA) was applied to determine group clustering trends and differential metabolites.

**Results:**

The results for the liver index, the liver function index and liver pathology showed that *Polygonum multiflorum* ethanol extract (PME), 50% ethanol elution fractions and tetrahydroxystilbene glucoside (TSG) from PME can induce idiosyncratic hepatotoxicity. TSG was the main idiosyncratically hepatotoxic component. Forty endogenous metabolites were identified in the rat liver. Six biomarkers, including lower levels of L-valine and higher levels of 3-hydroxybutyric acid, hexadecanoic acid, ribose, phosphoric acid and oxalic acid, were related to PM-induced liver injury. These differential biomarkers led to disruptions in amino acid, fatty acid, oxalate, energy and glucose metabolism. A total of 32 types of endogenous metabolites were identified in rat serum. Ten biomarkers were related to the liver injury induced by TSG, including lower levels of L-valine and L-proline and higher levels of urea, caproic acid, DL-malic acid, D-mannose, 3-hydroxybutyric acid, D-galactose, octadecane and hexadecanoic acid. These differential biomarkers led to disruptions in amino acid, glucose and fat metabolism. The mechanism of idiosyncratic hepatotoxicity in PM involves TSG-induced disruptions in amino acid metabolism, lipid metabolism, energy metabolism and glucose metabolism.

**Conclusions:**

These findings reflect the material basis and metabolic mechanism of idiosyncratic PM hepatotoxicity.

**Supplementary Information:**

The online version contains supplementary material available at 10.1186/s12906-021-03276-4.

## Background

*Polygonum multiflorum* Thumb. (PM) is widely used in traditional Chinese medicine and as a dietary supplement, but hepatotoxicity due to PM occurs in certain individuals. The biological effects of PM include detoxification, carbuncle elimination, bowel relaxation, malaria prevention, antiaging and hair blackening [1]. Although PM-induced liver injuries have increased significantly, some investigations of suspected clinical patients have revealed that such injuries occur in only a minority of patients and are related to idiosyncratic hepatotoxicity [2–5]. The hepatotoxicity of PM has also been summarized and recorded in the LiverTox® database, a comprehensive resource for idiosyncratic drug-induced liver injury (IDILI) produced by the National Institute of Diabetes and Digestive and Kidney Diseases and National Library of Medicine [4]. Although IDILI often occurs in a minority of patients (generally < 1%) [6], it is one of the leading causes of drug development failure and drug withdrawal from the market. IDILIs are often found after marketing or in the final phase of a clinical study since IDILIs cannot be evaluated in preclinical drug safety assessments using healthy animals [7]. Thus, animal models must be developed to assess drugs that cause IDILI. The inflammatory stress hypothesis has provided some of the first animal models of idiosyncratic hepatotoxicity in which nontoxic doses of IDILI-causing drugs are rendered hepatotoxic upon coexposure to a nontoxic but modestly inflammatory dose of bacterial endotoxin (lipopolysaccharide (LPS)) [8, 9]. The LPS model has been successfully used to evaluate several drugs known to cause IDILI in humans, including trovafloxacin, ranitidine, sulindac, chlorpromazine, halothane, monocrotaline, amiodarone and diclofenac [6]. In addition, combined treatment with a nontoxic dose of LPS and a therapeutic dose of PM resulted in acute idiosyncratic liver injury in rats [2]. In this study, the LPS model is used to evaluate the idiosyncratic hepatotoxicity of PM.

The components of PM include tetrahydroxystilbene glucoside (TSG), anthraquinone, phospholipids and tannins [10]. TSG, anthraquinone, tannins and biotoxins may be hepatotoxic components of PM [10, 11], but no definitive conclusion has been reached. Thus, the relationship between the hepatotoxic components and toxicity to the liver must be elucidated. Some scholars have reported the toxic effects of PM, but few have elucidated the components and metabolic mechanism underlying idiosyncratic hepatotoxicity. In this study, liver injuries in rats treated with *Polygonum multiflorum* ethanol extract (PME) and the effects of different elution fractions and TSG under nonidiosyncratic and idiosyncratic models were systematically investigated. The purpose of this study was to identify idiosyncratically hepatotoxic components and elucidate the metabolic mechanism of idiosyncratic hepatotoxicity due to PM. Gas chromatography/mass spectrometry (GC-MS)-based metabolomics was adopted to characterize PM-induced idiosyncratic hepatotoxicity and to explore the underlying mechanism. Unlike other functional genomic tools, metabolomics can provide a detailed and specific profile of the endogenous metabolic status of an organism in response to toxicological events. GC-MS is highly advantageous for detecting low-molecular-weight metabolites in metabonomic studies because GC-MS provides heightened equipment stability and user-friendly tools for data analysis. Untargeted metabolomics methods have been used to simultaneously detect several classes within the metabolome, allowing observation of changes in endogenous metabolites that are linked to toxicity. This study will provide an experimental basis for the primary hepatotoxic components in PM and elucidate the metabolic mechanism of hepatotoxicity.

## Methods

### Plant material

PM pieces were provided by the Hubei Yafei TCM Company (Batch number: 20170809) and were confirmed to be the dry root of *Polygonum multiflorum* by Limin Gong, an associate professor of the School of Pharmacy of Hunan University of Chinese Medicine.

### Materials and reagents

Chlorpromazine hydrochloride was obtained from Harvest Pharmaceutical Co., Ltd. (Shanghai, China). An alanine transaminase (ALT) kit and an aspartate transaminase (AST) kit were purchased from Nanjing Jiancheng Bioengineering Institute (Nanjing, China). A lactate dehydrogenase (LDH) kit was obtained from Wuhan Huamei Biotech Co., Ltd., China. Methoxyamine, N, O-bis (trimethylsilyl) trifluoroacetamide, pyridine, malic acid and LPS were purchased from Sigma Chemical Company (St. Louis, USA).

### Drug preparation

#### PME

A certain quantity of the PM powder (16 kg) was weighed, and three extractions of 2 h each were accomplished using eightfold, sixfold and sixfold dilutions of 70% ethanol. The extracts were combined and concentrated to dryness under a vacuum.

Different elution fractions of PME: portions of the PME were applied to macroporous resin and sequentially eluted with water, 50% ethanol and 95% ethanol. Each elution fraction was concentrated to dryness.

#### TSG

TSG was prepared in our laboratory. The structure was confirmed by comparing the compound’s ^1^H and ^13^C NMR spectra to reported spectra. Examination by HPLC showed that the purity reached 98% [12].

### Animals

Specific-pathogen-free (SPF) male rats (200 ± 20 g) were purchased from Hunan SJA Laboratory Animal Co., Ltd.; male rats are more susceptible to drug-induced idiosyncratic hepatotoxicity [5]. The animal permit number is SCXK (Xiang) 2016–0002. The temperature and humidity of the animal housing conditions met the requirements for housing experimental animals. The rats were kept under a 12-h dark-light cycle and were housed for one week prior to the experiments. Animal care and treatments were conducted according to established guidelines and protocols approved by Animal Care and Use Committee of Hunan University of Chinese Medicine (Changsha, China). All efforts were made to minimize the number of animals used and their suffering.

### Animal grouping and pharmacological intervention

According to the Chinese Pharmacopoeia, 3–6 g of raw PM is suggested for human clinical application. The corresponding clinical dose for a rat was determined to be 0.3125–0.625 g/kg. We selected the dose of PM according to this requirement, the actual administration volume and references [2]. We selected the dosages of the water elution fraction, 50% ethanol elution fraction and 95% ethanol elution fraction according to the yields of the different elution fractions of PM. The yields of the water elution fraction, 50% ethanol elution fraction and 95% ethanol elution fraction were 10, 20 and 10%, respectively. In addition, we selected the dose of TSG according to the percentage TSG content in PM (1%). We selected only one dose for the water fraction according to pre-experiment results, which showed that cotreatment with a nontoxic dose of LPS and water fraction (5.4 mg/kg) did not induce idiosyncratic hepatotoxicity.

A total of 240 male rats were randomly divided into 30 groups (*n* = 8 per group) assigned to the following treatments: the negative control (C); LPS model (M); chlorpromazine-positive group (P, chlorpromazine hydrochloride can cause hepatotoxicity through an inflammatory response [13, 14], 10.4 mg/kg); high-, medium- and low-dose PME (CPH 5.4, CPM 1.08 and CPL 0.54 g/kg, respectively); water elution fraction of PME (W 5.4 mg/kg); high-, medium- and low-dose 50% ethanol elution fractions of PME (FH 1.08, FM 0.216 and FL 0.108 g/kg, respectively); high-, medium- and low-dose 95% ethanol elution fractions of PME (NH 54, NM 10.8 and NL 5.4 mg/kg, respectively); high-, medium- and low-dose TSG (TSGH 108, TSGM 10.8 and TSGL 2.7 mg/kg, respectively); LPS + chlorpromazine-positive (PL); LPS + high-, medium- and low-dose PME (CPHL, CPML and CPLL, respectively); LPS + water elution fraction of PME (WL); LPS + high-, medium- and low-dose 50% ethanol elution fractions of PME (FHL, FML and FLL, respectively); LPS + high-, medium- and low-dose 95% ethanol elution fractions of PME (NHL, NML and NLL, respectively); and LPS + TSG high-, medium- and low-dose groups (TSGHL, TSGML and TSGLL, respectively). The groupings and acronyms for each group are shown in Table 1. The C and M groups were given normal saline by oral gavage each day, and the other groups were given 5-mL/kg aliquots by oral gavage. The M group and LPS + drug groups were injected with 2.8 mg/kg LPS at 4 h after drug administration each day [15]. Pharmacological intervention was carried out for 15 consecutive days. Livers were collected and weighed. Serum samples were prepared by centrifuging the collected blood samples (at 2000 rpm for 10 min at 4 °C) and then stored at − 80 °C. At the end of the treatment, rats were weighed and injected with a high concentration of pentobarbital sodium (80 mg/kg) for anesthesia, blood was collected from the abdominal aorta, and cervical dislocation was performed.

**Table 1:** The groupings and acronyms for each group

Groupings	Acronyms	Groupings	Acronyms
negative control (2.8 mg/kg)	C	LPS model (2.8 mg/kg)	M
chlorpromazine-positive (10.4 mg/kg)	P	LPS + chlorpromazine-positive (2.8 mg/kg + 10.4 mg/kg)	PL
high low dose PME (5.4 g/kg)	CPH	LPS + high dose PME (2.8 mg/kg + 5.4 mg/kg)	CPHL
medium dose PME (1.08 g/kg)	CPM	LPS + medium dose PME (2.8 mg/kg + 1.08 g/kg)	CPML
Low dose PME (0.54 g/kg)	CPL	LPS + low dose PME (2.8 mg/kg + 0.54 g/kg)	CPLL
water elution fraction of PME (5.4 mg/kg)	W	LPS + water elution fraction of PME (2.8 mg/kg + 5.4 mg/kg)	WL
high dose 50% ethanol elution fraction of PME (1.08 g/kg)	FH	LPS + high dose 50% ethanol elution fraction of PME (2.8 mg/kg + 1.08 g/kg)	FHL
medium dose 50% ethanol elution fraction of PME (0.216 g/kg)	FM	LPS + medium dose 50% ethanol elution fraction of PME (2.8 mg/kg + 0.216 g/kg)	FML
low dose 50% ethanol elution fraction of PME (0.108 g/kg)	FL	LPS + low dose 50% ethanol elution fraction of PME (2.8 mg/kg + 0.108 g/kg)	FLL
high low dose TSG of PME (108 mg/kg),	TSGH	LPS + high dose TSG of PME (2.8 mg/kg + 108 mg/kg),	TSGHL
medium dose TSG of PME (10.8 mg/kg)	TSGM	LPS + medium dose TSG of PME (2.8 mg/kg + 10.8 mg/kg)	TSGML
low dose TSG of PME (2.7 mg/kg)	TSGL	LPS + low dose TSG of PME (2.8 mg/kg + 2.7 mg/kg)	TSGLL

### Liver index and biochemical index assays

The liver index values were calculated using Eq. 1. The activities of ALT, AST and LDH were measured using the corresponding kits with a microplate reader (Thermo Electron Corporation, USA).

1 $$ \mathrm{Liver}\ \mathrm{index}\ \mathrm{values}=\left(\mathrm{liver}\ \mathrm{weight}/\mathrm{rat}\ \mathrm{weight}\right)\ast 100\% $$

### Hematoxylin and eosin (H&E) staining of idiosyncratic hepatotoxicity

The hepatic tissue samples were fixed in 10% formalin. Then, the paraffin-embedded tissue samples were sectioned into 5-μm-thick slices. The sections were stained with H&E and examined under a light microscope (Model IX71, Olympus, Tokyo, Japan).

### GC-MS-based hepatic and serum metabolomics study

#### GC-MS specimen pretreatment [16]

Liver homogenate/serum samples (100 μL) were thawed at 4 °C for 1 h and mixed with 50 μL of internal standard (1 mg/mL malic acid) by vortexing. After vortex mixing with 450 μL of methanol, each sample was allowed to settle for 8 min and then centrifuged at 13,000 RPM for 10 min. The supernatant was aspirated and dried under nitrogen. Methoxyamine pyridine (50 μL, 20 mg/mL) was added to the dried pellet, and the mixture was incubated for 1 h at 70 °C. Next, 100 μL of N, O-bis (trimethylsilyl) trifluoroacetamide was added to the solution, mixed for 30 s and then incubated at 70 °C for 1 h and at room temperature for 2 h. The sample was centrifuged at 13000 RPM for 8 min, and then 100 μL of the supernatant was transferred to a 250-μL internal tube that was placed into a sample vial for GC-MS analysis using a model QP2010 gas chromatograph-mass spectrometer (QP2010, Shimadzu, Japan).

#### GC-MS conditions [16]

A DB-5MS quartz capillary column (30 m × 0.25 mm × 0.25 μm, Agilent J&W Scientific, Folsom, USA) was used. The injector temperature was set to 280 °C, the injection volume was 1 μL, and the split ratio was 1:1. The flow rate of helium (carrier gas, 99.999%) was set to 1.0 mL/min. The column temperature was initially 70 °C, and this temperature was held for 4 min, gradually increased at a rate of 20 °C/min to 110 °C and then at a rate of 8 °C/min to 270 °C and then held for 5 min. The temperature of the electron ionization source was 200 °C. The solvent delay duration was 6.5 min, and the MS scanning range was 35–550 m/z. During the analysis, the sample injection sequence was randomly arranged, and one quality control (QC) sample was added every nine samples.

#### GC-MS data processing and analysis

Chromatograms were subjected to ion-pair extraction, peak alignment, peak matching and peak amplitude correction using XCMS software. A data set of the samples consisting of retention times and peak areas of metabolites was imported into SIMCA-P software (version 14.0, Umetrics AB, Umeå, Sweden) for orthogonal projections to latent structures discriminant analysis (OPLS-DA). Variables with importance parameter values (variable influence on projection, VIP) exceeding 1 in the OPLS-DA model were selected as potential differential metabolites. SPSS 13.0 statistical software (SPSS Inc., Chicago, USA) was used to perform *t*-tests. Metabolites with VIP > 1 and *P* < 0.05 (*t*-test) were considered statistically significant. Biomarkers were determined by comparison with biological databases, including the Kyoto Encyclopedia of Genes and Genomes (KEGG) and the Human Metabolome Database (HMDB).

### Statistical analysis

The data are represented as the mean ± SD and were analyzed by SPSS 13.0 software. Student’s *t*-tests were used for comparisons between groups. One-way analysis of variance (ANOVA) was used for comparisons among groups. Nonparametric data were analyzed by Mann-Whitney *U*-tests. *P* < 0.05 was considered statistically significant.

## Results

### Effects on the liver index

**Fig. 1:**
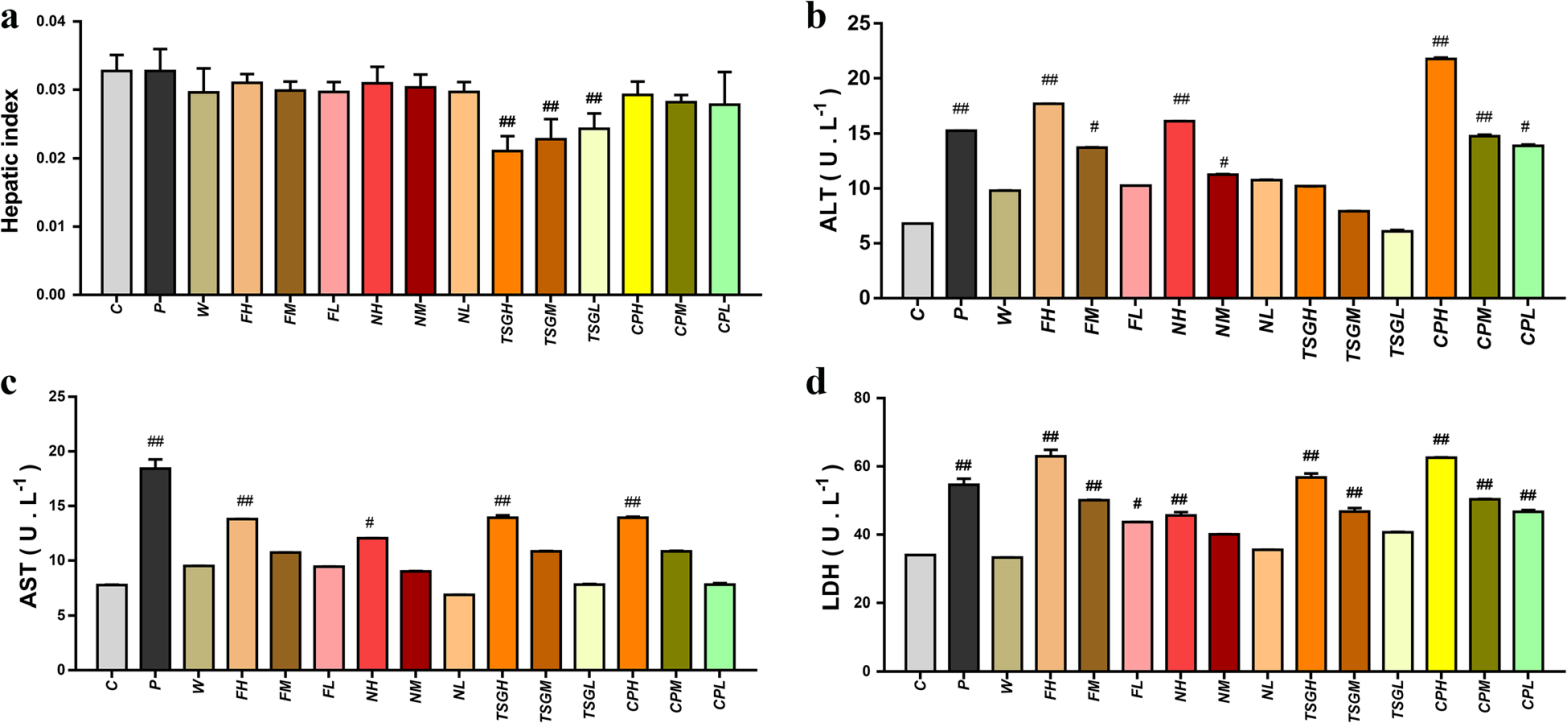
The effects of *Polygonum multiflorum* ethanol extract (PME), different fractions of PME and tetrahydroxystilbene glucoside (TSG) on the liver index and serum AST, ALT and LDH under nonidiosyncratic models. **a** Liver index. **b** The level of AST. **c** The level of ALT. **d** The level of LDH (D). ^##^: *P* < 0.01 compared to the C group, ^#^: *P* < 0.05 compared to the C group; Negative control group (C); Chlorpromazine-positive group (P); high, medium and low doses of PME (CPH, CPM and CPL, respectively); water elution fraction of PME (W); high, medium and low doses of the 50% ethanol elution fraction of PME (FH, FM and FL, respectively); high, medium and low doses of the 95% ethanol elution fraction of PME (NH, NM and NL, respectively); and high, medium and low doses of TSG (TSGH, TSGM and TSGL, respectively)

**Fig. 2:**
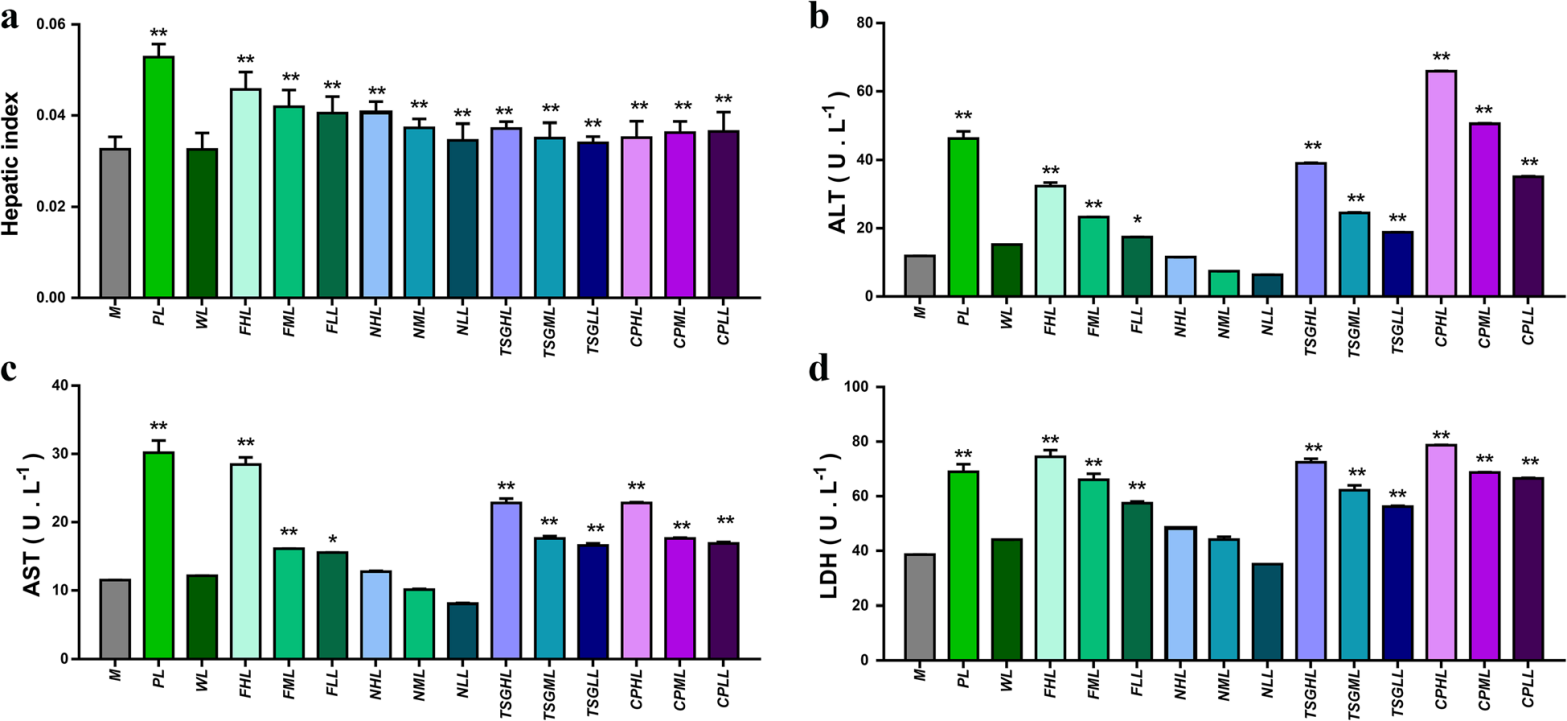
The effects of *Polygonum multiflorum* ethanol extract (PME), different fractions of PME and tetrahydroxystilbene glucoside (TSG) on the liver index and serum AST, ALT and LDH under idiosyncratic models. **a** Liver index. **b** The level of AST. **c** The level of ALT. **d** The level of LDH (D). **: *P* < 0.01 compared to the M group, *: *P* < 0.05 compared to the M group. LPS model group (M); LPS + chlorpromazine-positive (PL); LPS + high, medium and low doses of PME (CPHL, CPML and CPLL, respectively); LPS + water elution fraction of PME (WL); LPS + high, medium and low doses of the 50% ethanol elution fraction of PME (FHL, FML and FLL, respectively); LPS + high, medium and low doses of the 95% ethanol elution fraction of PME (NHL, NML and NLL, respectively); and LPS + TSG at high, medium and low doses (TSGHL, TSGML and TSGLL, respectively)

As a commonly used toxicology indicator, the liver index reflects liver toxicity. Compared to that in the control group, liver index values were significantly lower in the high-, medium- and low-dose TSG groups. The liver index values did not differ significantly in the other groups, and the results indicated that direct administration of PME, different elution fractions and TSG did not induce significant liver injuries (Fig. 1a). Compared with that in the model group, the liver index values in all the other groups except for the water elution fraction increased significantly in the LPS model (Fig. 2a). Direct administration of PME, the 50% ethanol elution fraction and TSG did not induce liver injury, but PME, the 50% ethanol elution fraction of PME and TSG administered with LPS caused liver injury. The results showed that the hepatotoxicity of PM was idiosyncratic.

### Effects on the liver function index

One of the key markers of liver damage is the release of enzymes such as AST, ALT and LDH into the circulatory system. As shown in Fig. 1b-d, compared to those in the C group, the levels of AST were significantly higher in the P, CPH, FH and TSGH groups (*P* < 0.01) and in the NH group (*P* < 0.05); the levels of ALT were significantly higher in the P, CPH, CPM, FH and NH groups (P < 0.01) and in the CPL, NM and FM groups (P < 0.05); and the levels of LDH were also significantly elevated in the P, CPH, CPM, CPL, FH, FM, NH, TSGH and TSGM groups (*P* < 0.01) and in the FL group (P < 0.05). As shown in Fig. 2b-d, compared to those in the M group, the levels of ALT, AST and LDH increased significantly in the PL, CPHL, CPML, CPLL, FHL, FHM, TSGHL, TSGML and TSGLL groups (P < 0.01). When the doses of TSG and PM increased, the levels of ALT, AST and LDH increased. The results indicated that TSG was the main idiosyncratically hepatotoxic component of PM, and the idiosyncratic hepatotoxicity of TSG was dose dependent. The levels of ALT were higher in the PME groups than in the TSG groups, and the results indicate that the other components may induce hepatotoxicity.

### Liver pathology examination

**Fig. 3:**
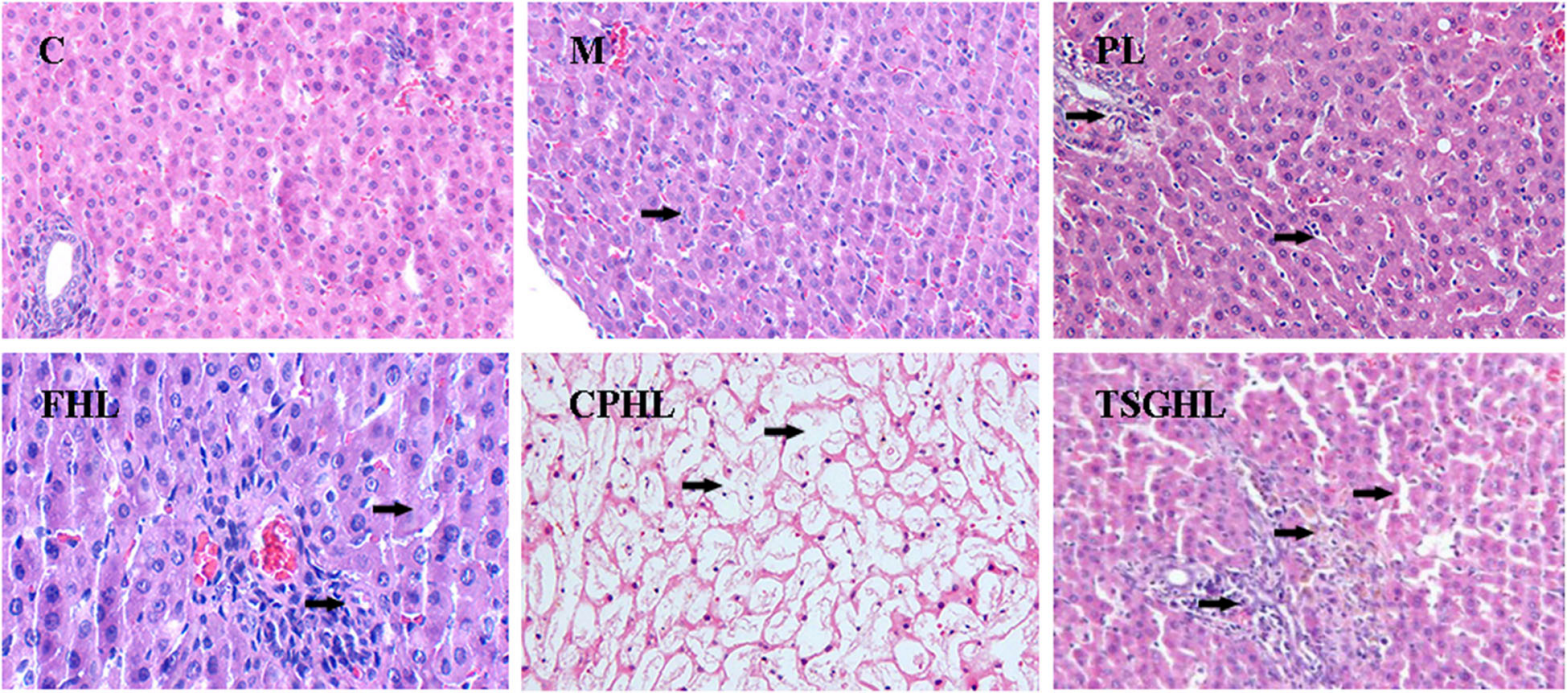
Comparison of the severity of rat liver injury (H&E staining, 200x, hepatic histological changes (arrow)). Negative control group (C); LPS model group (M); LPS + chlorpromazine-positive (PL); LPS + high dose of PME (CPHL); LPS + high dose of the 50% ethanol elution fraction of PME (FHL); LPS + high dose of the 95% ethanol elution fraction of PME (NHL); and LPS + TSG at high doses (TSGHL)

Direct administration of PME, different elution fractions of PME and TSG did not induce any significant pathological changes (Supplementary Fig. 1). The livers of the rats treated with LPS alone showed moderate infiltration of inflammatory cells. Severe liver injury was observed in the rats cotreated with LPS and the high dose of PME. The liver injuries included widespread hepatocyte necrosis, the disappearance of nuclei, moderate interstitial fibrosis and some infiltrated inflammatory cells. The nuclei of the hepatocytes were misshapen in the rats cotreated with LPS and the high-dose 50% ethanol elution fractions of PME. Hepatocyte necrosis was observed in the rats cotreated with LPS and the high dose of TSG (Fig. 3).

### GC-MS-based hepatic metabolomics study

**Fig. 4:**
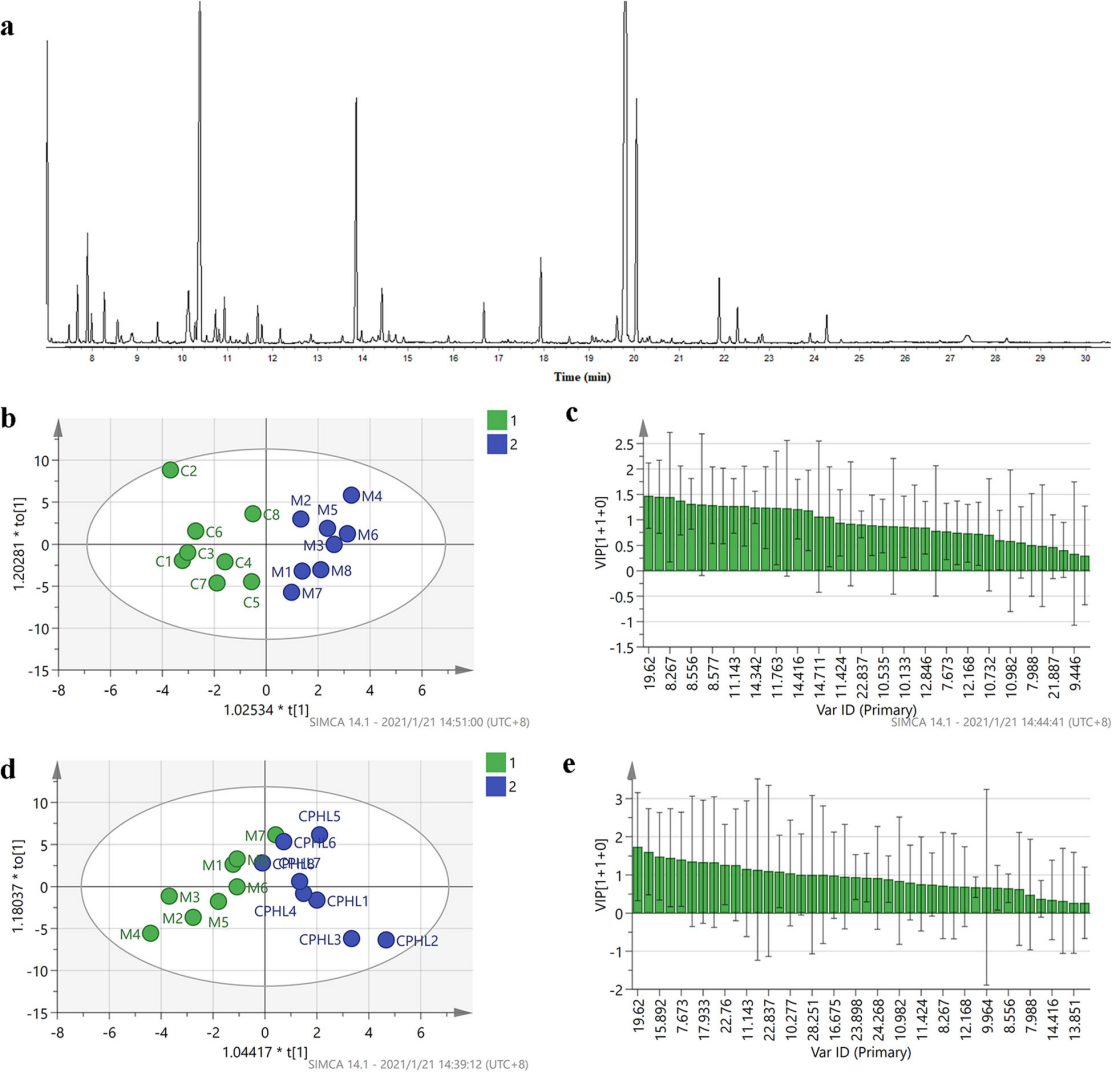
GC-MS total ion chromatograms and pattern recognition for OPLS-DA images of rat liver. **a** GC-MS total ion chromatograms of rat liver. **b** The OPLS-DA scores of the control group and the model group (1 C, 2 M). **c** The VIP values of differential markers of C and M. **d** The OPLS-DA scores of M and CPHL (1 M, 2 CPHL). **e** The VIP values of differential markers of M and CPHL

To clarify the mechanism underlying idiosyncratic PM hepatotoxicity, the metabolites showing differences between the negative control group and the LPS model group were identified and analyzed, and the differential metabolites between the M group and the CPHL groups were identified and analyzed. A total of 40 types of endogenous metabolites, including amino acids, carbohydrates, oxalate and fatty acids, were identified. The results are shown in Table 2 and Fig. 4a. The explanatory ability (given by the parameter R_2_Y) and the predictive ability (given by the parameter Q_2_) of the model established for the C and M groups were 0.836 and 0.463, respectively. The R_2_Y and Q_2_ of the model for the M and CPHL groups were 0.844 and 0.632, respectively. These results indicated that these models showed high degrees of differentiation and prediction.

**Table 2:** Hepatic differential metabolites and metabolic pathways in the different groups

No	t/min	Metabolites	m/z	Trend		Metabolic pathways
1	7.014	Propanoic acid	74.08			
2	7.417	l-Valine	117.15			
3	7.673	l-Alanine	89.09	↓^1)^	↓^2)^	Amino acid metabolism
4	7.988	Glycine	75.07	↑^1)^		Amino acid metabolism
5	8.267	Ethanedioic acid	90.03		↑^2)^	Oxalate metabolism
6	8.518	3-Hydroxybutyric acid	104.10		↑^2)^	Energetic metabolism
7	8.577	leucine	265.30			
8	8.894	l-Isoleucine	131.17			
9	9.964	Urea	60.06			
10	10.133	Serine	105.09			
11	10.277	Silanamine	80.59			
12	10.386	Silanol	90.20			
13	10.535	Hexadecane	260.65			
14	10.732	L-Threonine	119.12	↑^1)^		Amino acid metabolism
15	10.982	Glycine	75.07			
16	11.143	Butanedioic acid	118.09			
17	11.424	Pyrimidine	80.09			
18	11.667	2-Butenedioic acid	116.07			
19	11.763	Serine	105.09			
20	12.168	N, O, O-Tris (trimethylsilyl)-L-threonine	257.40			
21	12.846	l-Aspartic acid,	133.10			
22	13.541	Aminomalonic acid	119.08			
23	13.851	Malic acid	134.09			
24	13.968	Fructose benzoyl oxime	176.17			
25	14.342	L-Aspartic acid	133.10			
26	14.416	L-Proline	115.13			
27	14.576	2-Pyrrolidone-5-carboxylic acid	151.10			
28	15.892	Glutamine	169.11			
29	16.675	D-Ribose	150.13		↑^2)^	Glucose metabolism
30	17.933	Phosphoric acid	98.00		↑^2)^	Amino acid metabolism
31	19.62	D-Mannose	182.17	↑^1)^		Glucose metabolism
32	19.845	D-Glucose	180.16	↑^1)^		Glucose metabolism
33	20.062	D-Galactose	180.16			
34	21.887	Hexadecanoic acid	256.42			
35	22.29	Inositol	180.16			
36	22.76	D-Mannitol	182.17			
37	22.837	Octadecane	254.49			
38	23.898	Oleic acid	282.46			
39	24.268	Octadecanoic acid	284.48			
40	28.251	Hexadecanoic acid	256.42		↑^2)^	Fatty acid metabolism

Notes: LPS model group (M) vs. negative control group, ^1)^ *p*<0.01, lipopolysaccharide (LPS) + PM high dose group (CPHL) vs. LPS model group, ^2)^ *p*<0.01. ↑-increase, ↓-decrease. Negative control group (C)

As shown in Fig. 4b-c, the hepatic metabolites in the M group were significantly different from those in the C group. The hepatic metabolites in the M group were significantly different from those in the CPHL groups. The differential metabolites were screened based on VI*P* values from the OPLS-DA model (Fig. 4d-e). Variables with VIP values > 1 were considered critical for classification. The screened differential metabolites were subjected to significance tests, and variables with P values < 0.05 were considered to be associated with the idiosyncratic model and idiosyncratic hepatotoxicity. Compared to the C group, five variables involving glucose metabolism and amino acid metabolism were different in the M group, namely, reduced levels of L-valine, L-threonine, mannose and glucose and an elevated alanine level. Compared to the M group, the CPHL group showed one decreased variable (L-valine) and five increased variables, namely, 3-hydroxybutyric acid, hexadecanoic acid, ribose, phosphoric acid and oxalic acid. These hepatic metabolite changes showed that PM led to disruptions in the metabolism of amino acids, lipids, oxalate, energy and glucose. These results suggest that the mechanism of PM idiosyncratic hepatotoxicity involves disruption of amino acid, lipid, oxalate, energy and glucose metabolism.

### GC-MS-based serum metabolomics study

The results for the liver index, liver function index and liver pathology suggested that hepatotoxicity was idiosyncratic for high-dose PME, the 50% ethanol elution fractions of PME and TSG. Therefore, in this study, differential metabolites were identified between the control group and the LPS model group. High-dose PME, the 50% ethanol elution fraction of PME and TSG were able to induce serious idiosyncratic hepatotoxicity; thus, the differential metabolites were identified between the M group and the CPHL, FHL and TSGH groups.

**Fig. 5:**
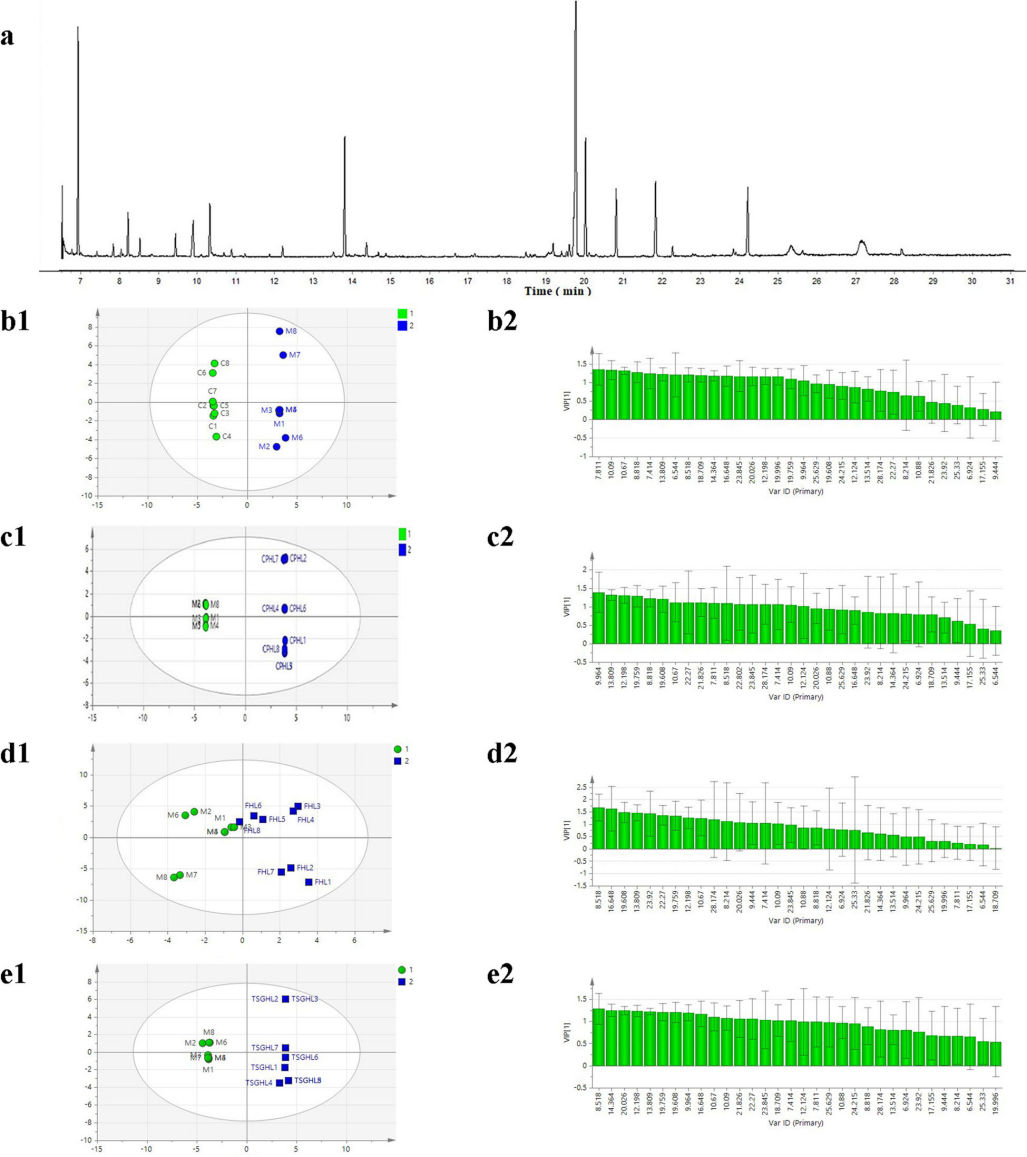
GC-MS total ion chromatograms and pattern recognition for OPLS-DA images of rat serum. (**a**) GC-MS total ion chromatograms of rat serum. (**b1**) The OPLS-DA scores of the control group and the model group (1 C, 2 M). (**b2**) The VIP values of differential markers of C and M. (**c1**) The OPLS-DA scores of M and CPHL (1 M, 2 CPHL). (**c2**) The VIP values of differential markers of M and CPHL. (**d1**) The OPLS-DA scores of M and FHL (1 M, 2 FHL). (**d2**) The VIP values of differential markers of M and FHL. (**e1**) The OPLS-DA scores of M and TSGHL (1 M, 2 TSGHL). (**e2**): The VIP values of differential markers of the M and TSGHL groups. Negative control group (C). LPS model group (M). LPS + PM high-dose groups (CPHL). LPS + 50% ethanol elution fraction high-dose groups (FHL) and LPS + TSG high-dose groups (TSGHL)

A total of 32 types of endogenous metabolites were identified, including amino acids, carbohydrates and fatty acids. The results are shown in Table 3 and Fig. 5a. The explanatory ability (given by the parameter R_2_Y) and the predictive ability (given by the parameter Q_2_) of the model established for the C and M groups were 0.880 and 0.574, respectively. The R_2_Y and Q_2_ values of the model for the M and CPHL groups were 0.966 and 0.894, respectively. The R_2_Y and Q_2_ of the model for the M and FHL groups were 0.788 and 0.561, respectively. The R_2_Y and Q_2_ of the model for the M and TSGHL groups were 0.789 and 0.532, respectively. These results indicate that these models featured high degrees of differentiation and prediction.

**Table 3:** Serum differential metabolites and metabolic pathways in the diffirent groups

No	t/min	Metabolites	m/z	Trend				Metabolic pathways
				M	CPHL	FHL	TSGHL	
1	6.544	4-Methylvaleric acid	116.16					
2	6.924	Propanoic acid	74.00					
3	7.414	L-Valine	117.15	↓^1)^	↓^2)^	↓^2)^	↓^2)^	Amino acid metabolism
4	7.811	Alanine	89.09	↑^1)^				Amino acid metabolism
5	8.214	Glycine	75.07			↑^2)^		Amino acid metabolism
6	8.518	3-Hydroxybutyric acid	104.11		↑^2)^	↑^2)^	↑^2)^	Energetic metabolism
7	8.818	L-Leucine	116.16	↓^1)^	↓^2)^			Amino acid metabolism
8	9.444	Methoxyacetlc acid	89.07					
9	9.964	Urea	60.06		↑^2)^	↑^2)^	↑^2)^	Microbial metabolism
10	10.09	L-Serine	105.09					
11	10.67	L-Threonine	119.09	↓^1)^				Amino acid metabolism
12	10.88	3-Aminoisobutyric acid	103.12					
13	12.124	Hexadecanoic acid	116.16		↑^2)^		↑^2)^	Glucose metabolism
14	12.198	Methoxyacetic acid	90.08					
15	13.514	DL-Methionine	131.17					
16	13.809	DL-Malic acid	134.09					
17	14.364	L-Proline	115.13	↓^1)^			↓^2)^	Amino acid metabolism
18	16.648	Ribitol	152.15					
19	17.155	Sulfurous	82.08					
20	18.709	Citric Acid	192.12	↓^1)^		↑^2)^	↑^2)^	Glucose metabolism
21	19.608	D-Mannose	180.16		↑^2)^	↑^2)^	↑^2)^	Glucose metabolism
22	19.759	D-Glucose	180.16		↑^2)^	↑^2)^		Carbohydrate metabolism
23	20.026	D-Galactose	180.16	↓^1)^	↑^2)^	↑^2)^	↑^2)^	Glucosemetabolism
24	21.826	Palmitic Acid	256.42					
25	22.27	Inositol	180.16					
26	22.802	Hexadecane	224.43					
27	23.845	Octadecane	254.49		↑^2)^		↑^2)^	Fatty acid biosynthesis
28	23.92	Oleic acid	282.46			↑^2)^		Amino acid metabolism
29	24.215	Octadecanoic acid	284.48					
30	25.33	Cholesterol	386.65					
31	25.629	Arachidonic acid	304.46					
32	28.174	Hexadecanoic acid	256.42			↑^2)^		Amino acid metabolism

Notes: LPS model group (M) vs. Negative control group, ^1)^ p<0.01, Lipopolysaccharide (LPS) + PM high dose group (CPHL), LPS + 50% ethanol elution fraction high dose group (FHL), LPS + TSG high dose groups (TSGHL) vs. LPS model group respectively, ^2)^ p<0.01. ↑-Increase, ↓-Decrease

As shown in Fig. 5 b1-e1, the serum metabolites in the M group were significantly different from those in the C group. Compared to those in the M group, the serum metabolites in the CPHL, FHL and TSGHL groups were significantly different. The differential metabolites were screened based on the VI*P* values of the OPLS-DA model (Fig. 5b2-e2). Variables with VIP values > 1 were considered to play critical roles in classification. The screened differential metabolites were subjected to significance tests, and variables with P values < 0.05 were thought to be associated with the idiosyncratic model and idiosyncratic hepatotoxicity. Compared to those in the C group, eight variables involving glucose metabolism and amino acid metabolism in the M group were altered, namely, reduced levels of L-valine, L-leucine, L-threonine, L-proline, citric acid, malic acid and D-galactose and an increased level of alanine. Compared to the M group, the CPHL group showed two decreased variables (L-valine and L-leucine) and seven increased variables, namely, the levels of D-mannose, D-galactose, D-glucose, 3-hydroxybutyric acid, urea, caproic acid and hexadecanoic acid. The FHL group showed nine differential variables, namely, lower levels of L-valine and L-threonine and increased levels of glycine, 3-hydroxybutyric acid, urea, D-mannose, fructose, D-galactose oleic acid and hexadecanoic acid. The TSGHL group showed 10 differential variables, namely, lower levels of L-valine and L-proline and higher levels of urea, caproic acid, DL-malic acid, D-mannose, 3-hydroxybutyric acid, D-galactose, octadecane and hexadecanoic acid. The serum metabolite changes showed that PME, the 50% ethanol elution fraction of PME and TSG all led to disruptions in amino acid metabolism, lipid metabolism, energy metabolism and glucose metabolism in the idiosyncratic model. In addition, TSG was the main chemical component of PME and the 50% ethanol elution fraction. These results suggest that the mechanism underlying idiosyncratic PME hepatotoxicity was that TSG led to disruptions in amino acid metabolism, lipid metabolism, energy metabolism and glucose metabolism.

## Discussion

PM is a traditional Chinese medicine and has been used in clinical practice and in the food industry for many years worldwide. However, the idiosyncratic hepatotoxicity of PM has attracted considerable interest. The 50% ethanol extract of PM (1.08 g/kg) may result in liver injury in the idiosyncratic model [2]. In this study, the results for the liver index, liver function index and liver pathology showed that the hepatotoxicity of PM was idiosyncratic. This result may explain why PM at clinically recommended safe doses and in treatment courses is not toxic to most individuals but is toxic to some individuals. TSG has two conformations, the cis and trans forms [15]. Furthermore, another study showed that trans-TSG can be photoisomerized to cis-TSG [17]. Cis-stilbene glucoside can induce immunological idiosyncratic hepatotoxicity [3]. Trans-TSG can increase the potential risk of liver injury from cis-TSG. In this study, a small portion of TSG may have been converted to cis-TSG during drug administration. This result is consistent with a previous report indicating that the toxicity of PM may be related to the content of tetrahydroxystilbene glucosides [12].

In general, the toxicity induced by a medication is proportional to the dose and the duration of exposure to the drug. Therefore, when the dose of TSG and PM increased, the levels of ALT, AST and LDH increased under nonidiosyncratic models in this study. This intrinsic hepatotoxicity mechanism was related to drug accumulation. Given that most people taking PM products at the recommended therapeutic dose do not develop liver injury, the intake of direct toxic components may not be sufficient to damage the liver but instead only initiates very mild hepatic cell stress, which can be resisted by the tissue regeneration, repair or antistress systems that maintain the normal structure and functions of hepatocytes exposed to low concentrations of toxic components [18]. In this study, direct administration of PME, different elution fractions of PME and TSG did not induce any significant pathological changes, which is consistent with the previous study [18]. Cotreatment with LPS at a nontoxic dose and PM at the clinically equivalent dose causes obvious liver injury in a dose-dependent manner. When the body is in a state of immunological activation, components (e.g., trans-TSG) with immunoenhancing activity might increase an individual’s susceptibility to potential toxic components (e.g., cis-TSG), leading to extensive injury of hepatic cells and overexpression of inflammatory cytokines [19]. An allergic constitution in a patient causes the metabolites and components of PM to be treated as haptens. After binding to their macromolecular carriers, the haptens form covalently bound whole antigens, which induce the production of antibodies and hypersensitivity [20]. In this study, the metabolites and components of PM and TSG may have induced immunological activation, immunoenhancing activity or haptens, which would promote idiosyncratic hepatotoxicity. Therefore, when the dose of TSG and PM increased, the levels of ALT, AST and LDH increased under idiosyncratic models. We think that idiosyncratic PM-induced liver injury is a result of complex interactions between drugs/reactive metabolites and the host immune response. However, the complex interactions between drugs/reactive metabolites and the host immune response must be clarified in further studies.

The high dose of PM (40 g/kg) altered lipid metabolism, amino acid metabolism and bile acid metabolism and excretion in a dose-dependent manner related to the mechanism of liver injury [21]. Cotreatment with a nontoxic dose of LPS and ethyl acetate extract resulted in clear liver injury, which mainly involved two pathways: tricarboxylic acid cycle and sphingolipid metabolism [22]. Rats treated with PM exhibited significant disturbances in energy metabolism and amino acid metabolism [23, 24]. In this study, liver injury in the rats treated with PME, different elution fractions and TSG under idiosyncratic models were systematically investigated. GC-MS-based hepatic and serum metabolomics was adopted to characterize PM-induced idiosyncratic hepatotoxicity and to explore the underlying mechanism. This study showed that the mechanism of PM-induced hepatotoxicity was based on disruption of energy metabolism and amino acid metabolism by TSG, explaining the mechanism of PM-induced hepatotoxicity. The liver plays important roles in fatty acid synthesis, *β*-oxidation metabolism and the maintenance of fatty acid levels, and 3-hydroxybutyric acid is an intermediate in fatty acid metabolism [25]. The increase in 3-hydroxybutyric acid indicated fatty acid metabolism disorder. Increased free fatty acid levels also indicated an abnormal tricarboxylic acid cycle and *β*-oxidation, suggesting that hepatotoxicity may be caused by hepatocytic mitochondria injury. The elevated levels of urea suggest that nitrogen-containing metabolites are increased. Nitrogenous wastes produced from protein and amino acid decomposition may induce toxicity.

## Conclusions

TSG was the main idiosyncratic hepatotoxic component, and liver injury increased with increasing TSG doses. A total of 32 types of endogenous metabolites were identified in rat serum. Ten biomarkers were related to the liver injury induced by TSG and led to disruptions in amino acid, glucose and fat metabolism. These findings provide the material basis and metabolic mechanism of PM-induced idiosyncratic hepatotoxicity.

## Supplementary Information

**Additional file 1: Supplementary Fig. 1.** Comparison of the severity of rat liver injury (H&E staining, 200x). Chlorpromazine-positive group (P); high, medium and low doses of PME (CPH, CPM and CPL, respectively); water elution fraction of PME (W); high, medium and low doses of the 50% ethanol elution fraction of PME (FH, FM and FL, respectively); high, medium and low doses of the 95% ethanol elution fraction of PME (NH, NM and NL, respectively); high, medium and low doses of TSG of PME (TSGH, TSGM and TSGL, respectively); LPS + medium and low doses of PME (CPML and CPLL, respectively), LPS+ water elution fraction of PME (WL); LPS + medium and low doses of the 50% ethanol elution fraction of PME (FML and FLL, respectively); LPS + medium and low doses of the 95% ethanol elution fraction of PME (NML and NLL, respectively); and LPS + TSG at medium and low doses (TSGML and TSGLL, respectively).

## Data Availability

All of the data analyzed in this study are included in this article.

## Abbreviations

PM: *Polygonum multiflorum*
PME: *Polygonum multiflorum* ethanol extract
TSG: tetrahydroxystilbene glucoside
LPS: Lipopolysaccharide
ALT: Alanine aminotransferase
AST: Aspartate aminotransferase
LDH: Lactate dehydrogenase
GC-MS: Gas chromatography/mass spectrometry
OPLA-DA: Orthogonal projection to latent structures discriminant analysis
VIP: Variable influence on projection
