# Supplementary material for: Investigation of the idiosyncratic hepatotoxicity of Polygonum multiflorum Thunb. through metabolomics using GC-MS
Source: BMC Complement Med Ther. 2021 Apr 12;21:120. doi: 10.1186/s12906-021-03276-4 (PMC8043067; doi:10.1186/s12906-021-03276-4)
Supplement: Supplementary file 1 — Additional file 1: Supplementary Fig. 1. Comparison of the severity of rat liver injury (H&E staining, 200x). Chlorpromazine-positive group (P); high, medium and low doses of PME (CPH, CPM and CPL, respectively); water elution fraction of PME (W); high, medium and low doses of the 50% ethanol elution fraction of PME (FH, FM and FL, respectively); high, medium and low doses of the 95% ethanol elution fraction of PME (NH, NM and NL, respectively); high, medium and low doses of TSG of PME (TSGH, TSGM and TSGL, respectively); LPS + medium and low doses of PME (CPML and CPLL, respectively), LPS+ water elution fraction of PME (WL); LPS + medium and low doses of the 50% ethanol elution fraction of PME (FML and FLL, respectively); LPS + medium and low doses of the 95% ethanol elution fraction of PME (NML and NLL, respectively); and LPS + TSG at medium and low doses (TSGML and TSGLL, respectively). [file 12906_2021_3276_MOESM1_ESM.docx]

**Supplementary Fig. 1**


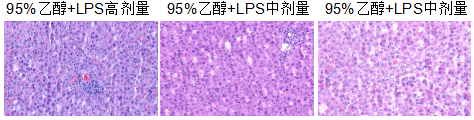

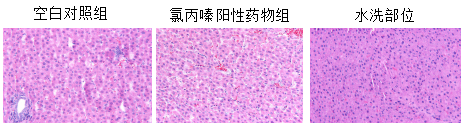

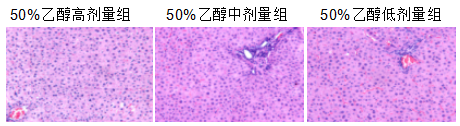


**P**

**W**

**FH**

**FM**


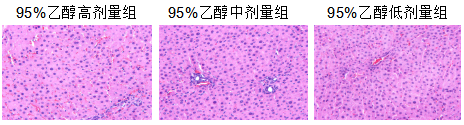

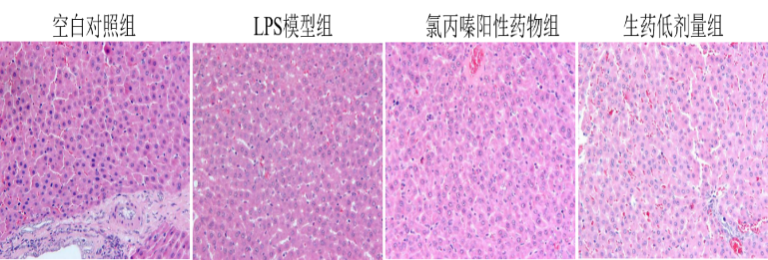

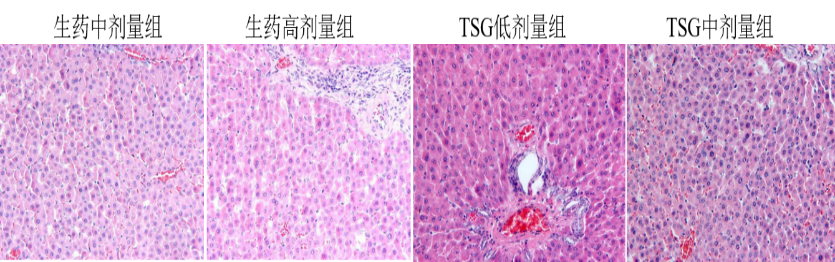


**NM**

**NL**

**CPH**

**CPL**


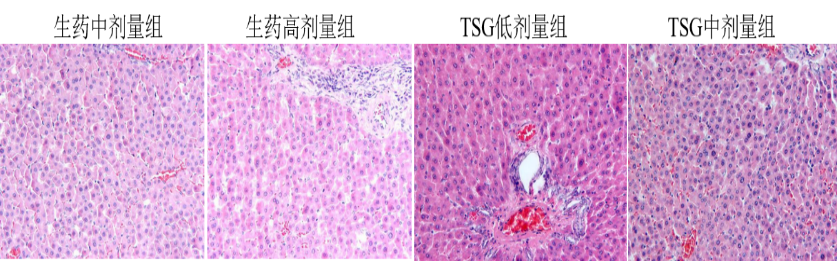


**CPM**


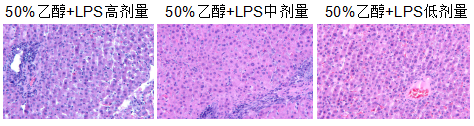

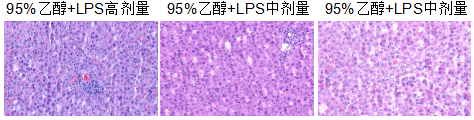


**NHL**

**FML**

**NLL**

**NML**


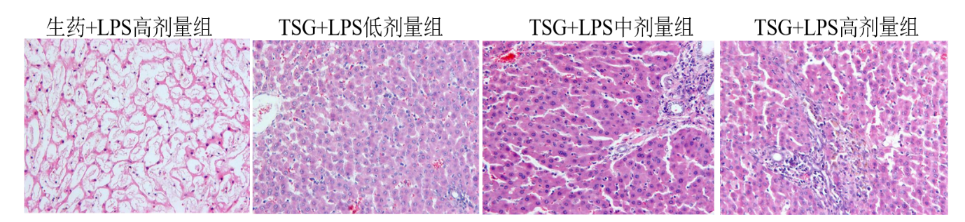

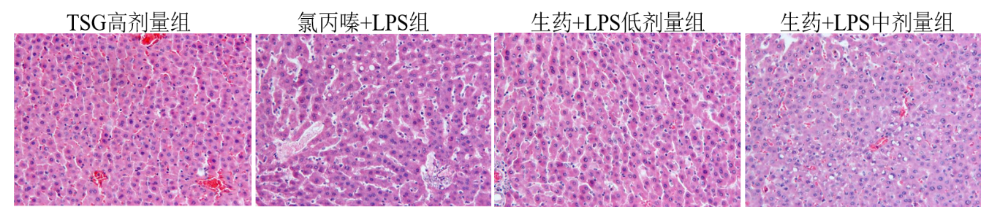


**TSGML**

**CPML**


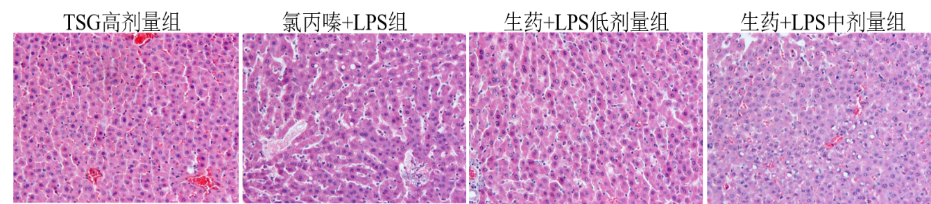


**CPLL**


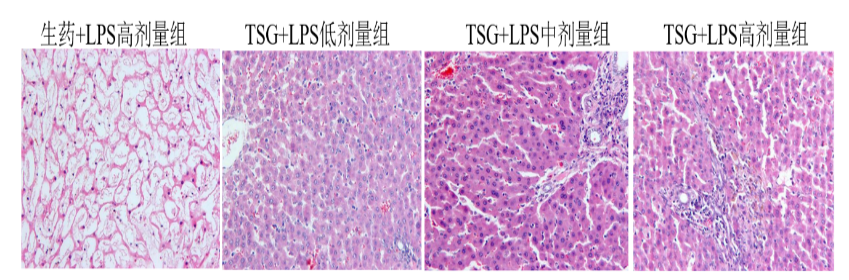


**TSGLL**


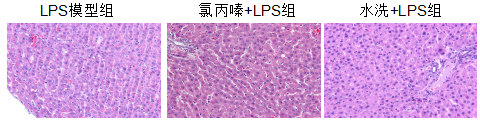

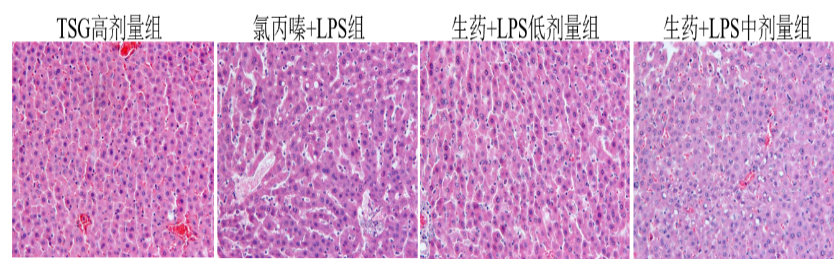

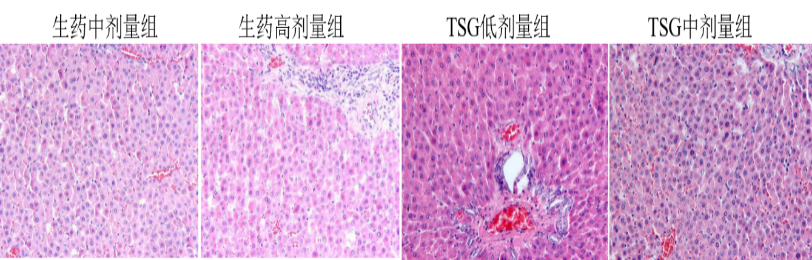


**TSGH**

**WL**


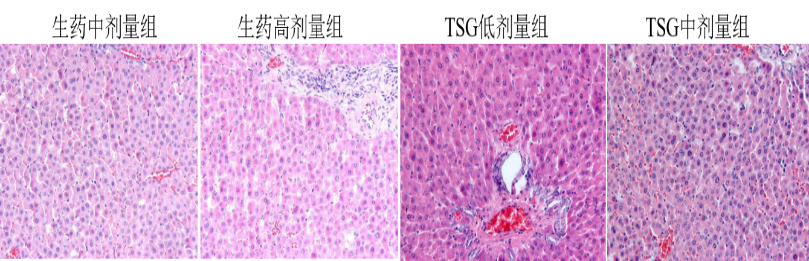


**TSGM**


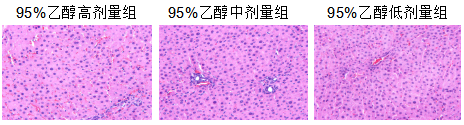


**NH**


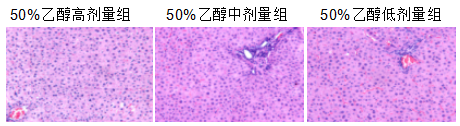


**FL**

**TSGL**

**FLL**
